# Towards a better understanding of the HTL process of lignin-rich feedstock

**DOI:** 10.1038/s41598-021-94977-w

**Published:** 2021-07-29

**Authors:** Benedetta Ciuffi, Massimiliano Loppi, Andrea Maria Rizzo, David Chiaramonti, Luca Rosi

**Affiliations:** 1 Chemistry Department “Ugo Schiff”, University of Florence, Via della Lastruccia 3-13, 50019 Sesto Fiorentino, Florence Italy; 2 RE-CORD, Viale Kennedy 182, Scarperia e San Piero, 50038 Florence, Italy; 3 Galileo Ferraris” Energy Department, Polytechnic of Turin, Corso Duca degli Abruzzi 24, 10129 Turin, Italy

**Keywords:** Analytical chemistry, Energy, Environmental chemistry

## Abstract

The hydrothermal liquefaction reactions (HTL) in subcritical conditions of a lignin residue has been studied on a lab scale. The starting material was a lignin rich residue co-produced by an industrial plant situated in Northern Italy producing lignocellulosic bioethanol. The reactions were carried out in batch mode using stainless steel autoclaves. The experiments were under the following operating conditions: two different temperatures (300–350 °C), the presence of basis catalysts (NaOH, and NH_4_OH) in different concentrations and the presence/absence of capping agent 2,6-bis-(1,1-dimethylethyl)-4-methylphenol (BHT). Lignin residue and reaction products were characterized by analytical and spectroscopic techniques such as CHN-S, TGA, GC–MS, EPR, and ^1^H-NMR with (2,2,6,6-Tetramethylpiperidin-1-yl)oxyl (T.E.M.P.O.). The addition of BHT did not significantly affect the yield of char which is formed by radical way. Spectroscopic analysis indicated that the level of radicals during the reaction was negligible. Therefore, the results obtained experimentally suggest that the reaction takes place via an ionic route while radical species would play a minor role.

## Introduction

The energy transition from fossil to renewable sources has created the need for a new paradigm in the exploitation of natural resources. In relation to this issue, biomasses such as lignin have aroused great interest. Lignin is the most abundant amorphous aromatic biopolymer on earth^1^. Together with cellulose and hemicellulose, it forms the secondary cell wall of plants, giving rigidity and mechanical strength to the cell. Lignin also promotes the transport of nutrients through the vascular bundles of the plant and acts as a protective barrier against parasites and pathogens^2^. The lignin content in plants is variable: in herbaceous species it is 15–20%, in soft woods 24–33%, while in hard woods it is 19–28%^1^. The chemical structure of lignin is very complex, as it is a highly branched and irregular phenolic polymer. As described by Lu et al.^1^, lignin is synthesized through a biosynthesis process which involves three basic phenylpropanoid monomers: p-hydroxyphenyl (H), guaiacyl (G), and syringyl (S). These in turn derive from alcoholic precursors: coumaryl alcohol, synapyl alcohol, and coniferil alcohol, all of which are generated by plant cells through a metabolic mechanism. The alcoholic precursors are connected to each other both through ethereal bonds (C–O–C) and carbon–carbon bonds (C–C). The main types of bonds between propionic units in soft and hard woods are: β–O–4 bonds (50%), β–β (5%), β–5 (10%), and α–O–4 bonds (10%)^3^. On an industrial level, lignin is obtained both as a by-product in the production of paper and cellulose pulp (black liquor)^4^ and as a waste product in bio refineries during the production of ethanol^5^. Currently the main use of by-product lignin is in energy enhancement in thermal cogeneration plants. This entails not only the loss of important aromatic resources that could be used for the production of chemicals or biofuels but, in the case of incomplete combustion, also the occurrence of serious environmental pollution problems^6^. An attractive, but as yet little exploited use of lignin is its enhancement through depolymerisation, with the aim of obtaining its constituent aromatic monomers. In recent years, amongst the different technologies being proposed for the depolymerisation of lignin, hydrothermal liquefaction (HTL) has been gaining increasing interest^7^. HTL is a thermochemical process which converts biomasses or other feedstock such as plastic waste into a liquid fraction, using water in supercritical (374.1 °C and 22.1 MPa) or subcritical conditions (temperatures below or close to critical point, and at pressures above saturation pressure)^8^.

An advantage of the HTL process is that by using water as a solvent, it can be used on materials with a high moisture content. The absence of a drying step therefore decreases the energy demand of the entire process.

The physicochemical properties of sub/supercritical water are very different from ambient liquid water. As an example, the dielectric constant trend is reported in Table 1.

**Table 1:** Variation of the dielectric constant of water with temperature and pressure^9^.

	Normal water	Subcritical water	Supercritical water
Temperature (°C)	25	350	400
Pressure (MPa)	0.1	25	25
Dielectric constant, * ε* (F m^−1^)	78.5	14.07	5.9

The dielectric constant (*ε*) of water at room temperature is approximately 78.5^9^. The dielectric constant is a parameter that determines the behaviour of the solvent and the ionic dissociation of the salts. A value of 78.5 makes water a fairly strong polar solvent. When water is heated and compressed up to the supercritical state, the dielectric constant drops to the typical values of a non-polar solvent, for example at 400 °C and 25 MPa, *ε* ⁓ 6^9^. This value is comparable to that of 1-dodecanol^10^. For these reasons supercritical water is an excellent solvent for non-polar substances such as organic compounds including lignocellulosic materials^11^.

It is a fact that the course of the reaction depends on the biomass used and the pre-treatment process used, but in any case, despite numerous existing studies on the depolymerisation of lignin with sub/supercritical water, a thorough understanding of the mechanisms involved is lacking. According to the literature, in fact, depolymerisation can proceed either by ionic (hydrolysis) or by a radical (pyrolysis) path. In the ionic mechanism, the main reactions involved are hydrolysis, demethylation, alkylation, and demethoxylation^12^. In particular, hydrolysis causes the breaking of the ether bonds, C–O–R, by water molecules. According to some authors^12–14^ the addition of basic substances such as Na_2_CO_3_, K_2_CO_3_, KOH, and NaOH to the reaction environment, promote a ionic pathway for the depolymerisation process. This happens because the presence of a carbonate or an alkaline cation polarizes the ethereal bond, thus shifting the reaction to the ionic cleavage of this bond. These reagents also show a catalytic function, suppressing not only the production of char but also increasing the oil yield and its quality. The effect of NaOH on the HTL of lignin was described by Roberts et al.^14–16^. According to their studies, the cleavage of the β–O–4 etheric bond occurs heterolytically via a six-membered transition state, a process by which the sodium cation and the hydroxide ions are also involved. Sodium cations form cationic adducts with lignin and polarize the ether bond. This leads to an increase in the negative partial charge on the oxygen atom, and reduces the energy required for the cleavage of the heterolytic bond. Moreover, they show that phenolic monomers are the primary products of lignin depolymerisation under basic conditions and subsequent oligomers are the result of consecutive condensation reactions between these monomers.

As previously mentioned, the other reaction mechanism suggested by some authors is the radical type, which involves an homolytic breaking of the bonds in the biopolymer. In particular, Yong and Matsumura^17,18^, studied the depolymerisation reaction of lignin in water, and conducted experiments under supercritical (390−450 °C, 25 MPa) and subcritical conditions (300−370 °C, 25 MPa). The results of these two studies were compared to each other to investigate the effect of temperature on lignin decomposition. The conversion of lignin under subcritical conditions occurs rapidly, despite a higher degree of depolymerisation being achieved under supercritical conditions. In supercritical conditions, the formation of char is very marked compared to the subcritical conditions. According to the authors this is irrefutable evidence that radical species are involved in the char formation mechanism. These radical species come from the breakdown of the β–O–4 bonds and their consequent re-polymerization through cross-linking forms higher molecular weight fragments, which give rise to the char. If the depolymerisation took place via a radical pathway, the addition of capping agents would greatly decrease the amount of the produced char. For example, according to the study of Pederson et al.^19^ on the hydrothermal liquefaction of biomass, glycerol can be used as a radical scavenger to reduce the yield of char. Another well-known capping agent described in literature is phenol^20^. Okuda et al.^21^, investigated the depolymerisation of lignin in a water–phenol mixture at 673 K. As hypothesized by the authors, the structure of lignin is decomposed by hydrolysis and dealkylation reactions. Subsequent cross-linking reactions are depressed due to entrapment of active fragments and capping of active sites by excess phenol. They revealed that the addition of phenol for lignin conversion in water is an effective method for conversion of lignin to lower molecular weight fragments. Recently some of us conducted in-depth studies of the HTL of lignin under subcritical conditions, in which we related operational parameters with the nature of the reactions products^22,23^. The aim of this present paper, however, is an attempt to understand the most likely reaction mechanism involved. Numerous experimental tests were conducted for this purpose. The knowledge of the reaction mechanism is of paramount importance, since it should allow us to drive the reaction towards the obtaining of specific products and to reduce the production of char, which negatively affects HTL reactions as it suppresses the production of the biocrude (in our text light oil/ heavy oil), which constitutes the desired product of the reaction.

## Results and discussion

### Feedstock

The properties of feedstock are described in great detail in our previous study^22^^.^ Table 2 shows the results obtained from the CHN-S and proximate analyses.

**Table 2:** CHN-S and proximate analysis of the feedstock.

Carbon%	54.2
Hydrogen%	5.9
Nitrogen%	1.0
Solfur%	0.2
Oxygen%	36.1
Ashes%	2.6
Volatile matter%	71.0
Fixed carbon%	26.4
Moisture%	69.7

The total lignin content of the feedstock, together with its residual structural sugars is reported in Table 3.

**Table 3:** HPLC analysis of the feedstock slurry.

Insoluble lignin%	57.50
Soluble lignin%	0.27
Total lignin%	57.80
Structural sugars%	35.46
Glucan%	3.10
XMG (xylose-mannose-galactose)%	5.13
Arabinose%	0.20

### Yields

**Figure 1:**
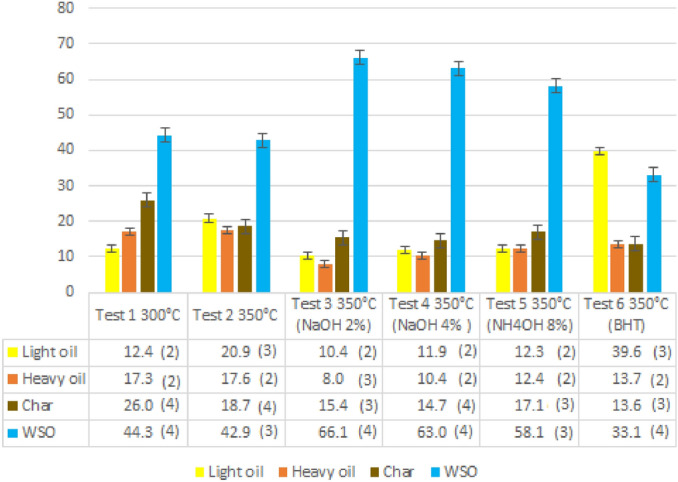
Yields of light oil, heavy oil, char and WSO. The semi-dispersion is shown in brackets.

For each test, the yields of light oil, heavy oil, char, and WSO were reported in Fig. 1.

The yields for test 7 were not calculated as the radical adducts with T.E.M.P.O. are very labile and the time required to calculate the yields would have favoured their recombination. Therefore, priority was given to the ^1^H-NMR analysis.

It can be observed that higher temperatures (350 °C) give higher yields in light oil. Comparing test 1 with test 2, the light oil yield increases from 12.4 to 20.9%, while the char yield decreases (from 26.0 to 18.7%). On the other hand, the heavy oil yield increases only slightly, suggesting that an increase in temperature favors the formation of lower molecular weight compounds. The addition of a base sharply favors the formation of water-soluble compounds (from 42.9% in test 2, with no base, to 66.1% in the presence of NaOH in test 3). Interestingly, however, when increasing the amount of base (test 4, NaOH to 4%), no benefit in yields was observed. The strength of the base did not significantly affect the yields of the products as can be observed when comparing test 3 (NaOH) with test 5 (NH_4_OH). In the sixth test, with BHT, the yields in char and heavy oil are in line with those of previous tests. The high yield of light oil is due exclusively to the large amount of BHT placed in the autoclave at the beginning of the reaction and consequently undergoing HTL in the same way as the feed. During our preliminary study we have not conducted specific tests on the stability of BHT in subcritical conditions, but we have assumed that its possible decomposition products, being phenolic in nature, they will be distributed in the oily phase and still act as capping agents.

### Test with BHT

As previously mentioned, some authors have suggested a radical path explanation for the hydrothermal liquefaction reaction of lignin. During the reaction, the radical fragments apparently couple together to produce char. In order to verify this hypothesis, we carried out a test in the presence of BHT. BHT is a radical capping agent with proven efficiency^24^ commonly used in industry as a food and cosmetic preservative, but also in pharmaceuticals, petroleum products, and rubbers^25^. BHT is able to suppress the radical species present in the reaction environment, through the homolytic cleavage of the O–H bond and through the donation of a hydrogen radical. Once formed, the BHT radical cannot react further because of the large steric hindrance created by the tert-butyl groups. These tert-butyl groups prevent the oxygen atom which carries the unpaired electron density from reacting with other molecules. If char is formed in a radical way, in the presence of BHT, a decrease of char yield should be observed in relation to the test without BHT. In the test performed in the presence of BHT, the yield of char did not decrease, still ranging from 12–14%, in line with tests conducted without the presence of BHT. This evidence suggests that, in the well-defined experimental conditions herein adopted, the absence or presence, or slight presence, of radicals are not primarily responsible for the formation of char.

### The effect of changing the nature of the ions on the composition of the light oil

The composition of the light oils obtained were determined by GC–MS analysis. Table 4 shows a comparison between the composition of the light oil obtained in test 3 and the light oil obtained in test 5. The aqueous phase of test 3 had an initial pH value of 12.0 and a final value of 8.8. In test 5 the initial pH was 11.9, while the final pH was 10.8.

**Table 4:** Comparison in the composition of light oils obtained in test 3 and in test 5.

Composition	Test 3 (NaOH 2%) µg/mL	Test 5 (NH_4_OH 8%) µg/mL
Phenol	129.15	41.08
m/p-Cresol	20.39	20.58
Guaiacol	36.02	28.81
Creosol	23.11	23.39
1,2-benzenediol-3-methoxy	31.75	0.00
Catechol	0.00	77.18
p-ethyl-guaiacol	23.18	19.01
1,2-benzenedio-4-methyl	0.00	100.00
Syringol	25.17	24.28
4-propylguaiacol	33.67	0.00
4-ethylcatechol	0.00	100.21

There are some remarkable differences in the composition of the two oils. The concentration of phenol in an ammonia environment becomes about one third of that in a basic environment determined by sodium hydroxide. Futhermore, catechol, 4-ethylcatechol, and 1,2-benzendiol-4-methyl were detected exclusively in the ammonia environment. Conversely, in the presence of NaOH, 1,2-benzendiol-3-methoxy and 4-propylguaiacol are present, but they are absent in the ammonia environment. The fact that different compounds are formed in the presence of different ions (Na^+^ and NH_4_^+^), suggests that ions play a fundamental role in the reaction mechanism, which we hypothesize takes place via an ionic pathway. The high concentration of catechols in the ammonia environment is a potentially useful finding. Catechols are chemicals that can be used as precursors for valuable chemicals, for example perfumes, pharmaceuticals or as building blocks in organic synthesis and as such are of high interest to various industries.

### EPR

The EPR analysis was performed on the light oil obtained in test 3, in order to detect the presence of radical species with a long life time. The oil was extracted in diethyl ether and stored at a temperature of − 8 °C before the analysis.

**Figure 2:**
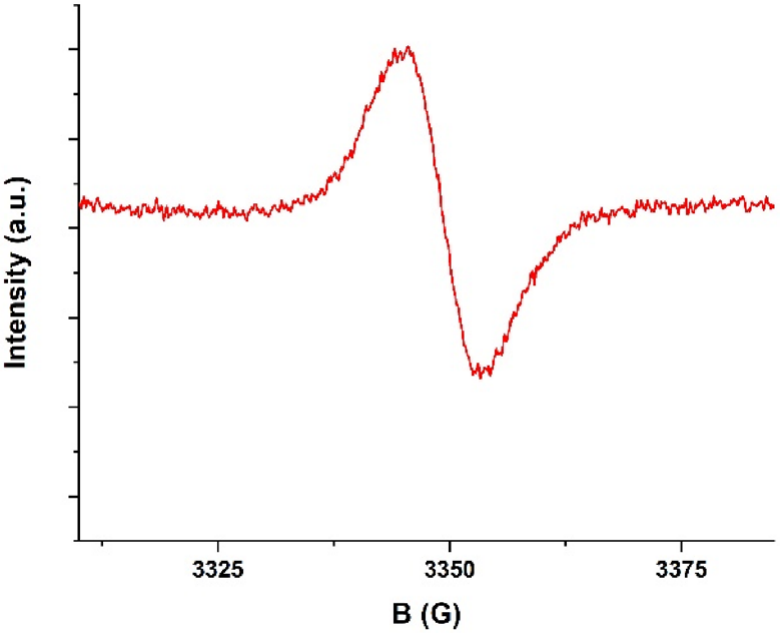
EPR spectrum of light oil obtained from test 3.

The EPR spectrum (Fig. 2) shows a single linear signal, perfectly symmetrical and devoid of fine structure. The spectral peak-to-peak width (∆H_p–p_) and g-factor are 8.17 G and 2.005 respectively. The g factor of an EPR spectrum can be used to establish what type of radical is involved. If radicals are centered on carbon, they have g value closer to that of the “free electron” (2.0023). Radicals centered on a carbon atom adjacent to an oxygen atom have a higher g value in the range between 2.003–2.004, while O-centered radicals have g values ​​higher than 2.004^26^. Specifically, a g value of 2.005 is a characteristic of phenolic radicals (phenoxyl radical)^27^. The presence of a single signal suggests that in the analysed sample there are no other radical species, with long life time, other than the one identified, so we can exclude the idea that the radical pathway is the main depolymerization route. These results are in agreement with what is described by Lyckeskog^28^. She found that during the depolymerization reaction of lignin, in the presence of a base, and at high temperatures, demethylation reactions prevail, leading to an increase in compounds with –OH groups such as phenols and catechols and to a decrease in the –OCH_3_ groups (guaicols).

It is important to underline that the observation of a single radical species does not exclude the presence of other radicals with a very short life time, which are likely to recombine immediately, and are thus difficult to detect by means of EPR technique. To understand if this type of species were present, we conducted a ^1^H-NMR analysis with a radical trap T.E.M.P.O.

### ^1^H-NMR with T.E.M.P.O.

**Figure 3:**
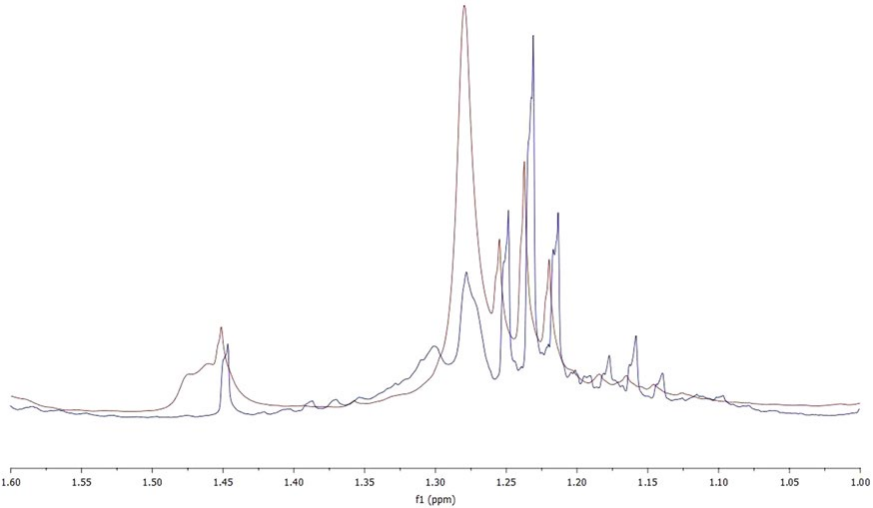
^1^H-NMR spectra of light oil from test 3 (red) and test 7 (blu).

**Figure 4:**
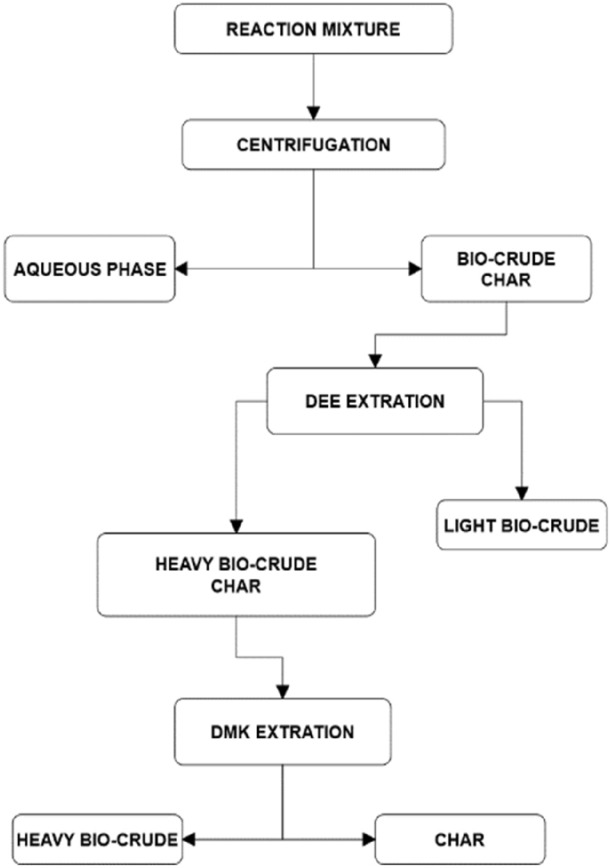
Procedure for separating the reaction products.

T.E.M.P.O. is a stable radical that exists as a red–orange solid at room temperature. The stability of this radical can be ascribed largely to resonance, while its persistence, largely to steric effects^29^. Thanks to these properties, it is used as a radical trap in EPR and NMR spectroscopies. In the presence of radicals, it forms an adduct that can be separated in TLC and analysed. In this study, we conducted a test ​​using T.E.M.P.O, and the light oil thus obtained was analysed via ^1^H-NMR. The spectrum obtained (Fig. 3, blue) was compared with that obtained from the light oil deriving from test 3 which had no marker (Fig. 3, red). The signals of the hydrogen of the methyl groups of T.E.M.P.O. are expected to fall in the region between 1.5 and 1 ppm. However, if the marker had formed an adduct with a radical species present in the sample, the methyl signals would have had to undergo a shift. As can be seen from Fig. 4, there is a complete superimposition of the two spectra which indicates a lack of adduct formation.

Comparing the data obtained in this test with those deriving from the EPR analysis, we can reasonably assume that there were no radicals with a short life time in the sample.

### Considerations on heating rate (HR)

As described in literature, the variation in HR can modify the concentration of radicals produced during the reaction^30^. The maximum concentration of radicals occurs with low HR, while with higher heating rates, their concentration decreases. We can therefore assume that the radical species observed in the EPR analysis are exclusively due to the secondary reaction caused by the low HR imposed by the instrumental limits.

## Conclusion

The aim of this work was to discuss which mechanism of depolymerisation of lignin in subcritical water was the most likely. Our experimental tests have shown that the use of BHT, a well-known inhibitor of radicals, did not lead to evident variations in the yield of char. The EPR and ^1^H-NMR spectroscopic analyses did not show a relevant presence of radicals. Moreover, the addition of ions of different kinds, resulted in the creation of different reaction products and this event is only possible if the reaction goes through an ionic path. These considerations suggest that, under the experimental conditions adopted, an ionic mechanism can be reasonably hypothesized. No doubt other studies are needed to corroborate our hypothesis.

## Materials and methods

### Feedstock

The lignin-rich feedstock used in the experiments was a bio-residue obtained after biomass-to-ethanol process from an industrial plant located in Northern Italy. The original feedstock used in the ethanol biorefinery was poplar wood. The feedstock was received in the form of wet agglomerated particles, with 69.7% by weight moisture content. The material was then dried, knife-ground and sieved through a < 0.25 mm mesh before being used in the tests. Carbon, hydrogen, nitrogen, and sulfur content (CHN-S), were quantified using a Leco (St. Joseph, MI, USA) TruSpec (UNI EN 15104, ASTM D4239) and oxygen was obtained by difference. The moisture content, ash content, and volatile matter were determined in a Leco TGA 701 (UNI EN 13040, UNI EN 14775, UNI EN 15148), and fixed carbon was also calculated by difference. The feedstock was further characterized after mixing it with water for a slurry preparation, quantifying the water-soluble compounds (WSO) in a LC–20 AT Prominence (Shimadzu, Kyoto, Japan) prior to the hydrothermal reactions. The HPLC apparatus is equipped with a refractive index detector, a Hi-Plex H (300 × 7.7 mm) column, and a PL Hi-Plex H (50 × 7.7 mm) guard column (Agilent, Santa Clara, CA, USA), operating at 55 ^◦^C with a flow of 0.6 mL∙ min^−1^ with 0.005 M sulfuric acid as the mobile phase. Moreover, the percentage of lignin and structural sugars were determined according to the NREL/TP–510–42618 procedure^31^.

### Experimental equipment and procedure

The hydrothermal liquefaction experiments were performed in an electrically heated 160 mL stainless steel Parr autoclave. The autoclave was equipped with a stirrer, a pressure sensor (model Parr 4842, in which pressure is displayed with 1 psi resolution and 10 psi accuracy), and a J-type thermocouple. The heating system consisted of a 1 kW electric band heater regulated by a PID controller. Overall seven tests were carried out in duplicate. The operating conditions are reported in Table 5.

**Table 5:** Operating conditions for each experiment.

ID #	Temperature (°C)	Reaction time (min)	Liquid solid ratio	Catalyst	Additive
1	300	10	10	–	–
2	350	10	10	–	–
3	350	10	10	NaOH 2%	–
4	350	10	10	NaOH 4%	–
5	350	10	10	NH_4_OH 8%	–
6	350	10	10	NaOH 2%	BHT
7	350	10	10	NaOH 2%	T.E.M.P.O

For each test, 7 g of lignin was used in combination with 70 mL of total liquid volume, consisting of ultrapure water (0.05 µS∙cm^−1^) or ultrapure water + basic solutions. This mixture, or mixture plus additive/s, was transferred to the Parr autoclave, which was then closed. Before each experiment, a leakage test over the autoclave with Argon at 80 bar was performed, and then subsequently, three purging cycle with N_2_ (5 bar) were carried out in order to ensure an inert atmosphere. Finally, the autoclave was charged with 6 atm of N_2_. In all tests except the first one, the temperature was set at 350 °C, and the average heating rate was 4.4 °C ∙ min^−1^. The reaction temperature was reached in 75 min and was maintained for 10 min. At the end of the reaction, the autoclave was rapidly cooled by immersing it in a bath of water and ice, and the gas was vented out. The reaction products were collected by means of the procedure reported in Fig. 4. The aqueous phase containing soluble organic compounds (WSO) was separated at the end of the cooling process via centrifuge and decanting. The reactor and its contents were washed with diethyl ether (DEE), and then filtered under vacuum over a glass microfiber filter (1 µm). The DEE-insoluble was washed with acetone (DMK) and filtered under vacuum. The solid residue in the DMK was then dried. The oil extracted in DEE was called *light bio-crude* or simply *light oil*, while the oil extracted DMK was called *heavy bio-crude* or simply *heavy oil.*

In the first and second tests no catalysts and additives were used. The third test was performed in a basic environment, adding a 1 M solution of NaOH till a 2 wt.% mixture. The fourth test (ID 4) was conducted in the presence of an even higher percentage of NaOH (4% wt.). In the fifth test, the nature of the base was changed, adding a solution of NH_4_OH in order to reach a pH similar to that of the third test. In the sixth test, in addition to the NaOH solution, 3.5 g of BHT was added as a radical scavenger. Finally, in the seventh test T.E.M.P.O. was added in the reaction environment as radical trap for the subsequent analysis of ^1^H-NMR.

### Determination of the yields

For each test, the yields of solid residue and oil products were determined using the equations^23^ below:

1 $${Y}_{Solid}\left(\%\right)=\frac{{W}_{Solid residue}}{ {W}_{Feedstock}}\times 100$$

2 $${Y}_{Oil}\left(\%\right)=\frac{{W}_{Oil}}{ {W}_{Feedstock}}\times 100$$

The yields were on a water-free basis.

The formation of gaseous products was not detected in any of our tests, as the final pressure registered by the pressure gauge was equal to the pressure measured at the start of the test. This means that or the pressure was below the detection limit of the instrument or no gases have formed.

Known the yields in light oil, heavy oil and char, the yields in WSO were calculated by difference. A TOC analysis was then performed on the aqueous phases in order to more accurately estimate the organic carbon present in each phase.

### Analytical methods and chemicals

Qualitative and quantitative analysis of the organic compounds in the oil were performed by GC–MS, equipped with a Zebron ZB-5HT INFERNO (Phenomenex) column (length 30 m, internal diameter 0.250 mm, film diameter 0.25 μm).

About 0.1 g of oil was dissolved in 10 mL of dichloromethane and then 2 µL of this solution was injected into a GC–MS apparatus (GC 2010 with a GCMS-QP2010 mass spectrometer, Shimadzu). The analysis was performed with a helium column flow of 2.02 mL∙ min^−1^, with an initial temperature of 40 °C (holding time 10 min) increased to 200 °C (heating rate 8 °C min^−1^, holding time 10 min), and then to 280 °C (heating rate 10 °C min^−1^, holding time 30 min). The GC–MS apparatus was used to determine the qualitative composition of the sample by comparing the acquired spectra against those of the NIST 17 library. For the TOC analysis, the 50–800 mg/L Spectroquant kit with TR 320 microreactor was used. Before proceeding with our analysis, a calibration line was made with 4 concentration points (50, 100, 200, 500 mg/L) and the analyzed samples were diluted to fall into the above range. The EPR spectrum of light bio-crude (test 3) was acquired in X-band, at room temperature, in a diethyl ether solution, using the EPR E500 Bruker Elexys spectrometer, equipped with a CF helium cryostat from Oxford Instruments (4–300 K). Glass silica capillaries were used for the insertion of the sample into the resonant cavity. The ^1^H-NMR spectra on light oil (test 7) were recorded in CDCl_3_ using Varian Mercury Plus 400 operating at 400 MHz at room temperature. NMR signals refer to not deuterated residual solvent signals (7.26 ppm for ^1^H). Chemical shifts (*δ*) are given in parts per million (ppm).
